# Piceatannol Attenuates Testosterone-Induced Benign Prostatic Hyperplasia in Rats by Modulation of Nrf2/HO-1/NFκB Axis

**DOI:** 10.3389/fphar.2020.614897

**Published:** 2020-12-21

**Authors:** Basma G. Eid, Ashraf B. Abdel-Naim

**Affiliations:** Department of Pharmacology and Toxicology, Faculty of Pharmacy, King Abdulaziz University, Jeddah, Saudi Arabia

**Keywords:** piceatannol, HO-1, Nrf-2, benign prostatic hyerplasia, rats

## Abstract

Benign prostatic hyperplasia (BPH) is a serious illness affecting middle-aged and elderly male patients. It is a complication of several diseases including metabolic syndrome. BPH has been associated with inflammation and increased oxidative stress in prostatic tissues. Piceatannol (PIC) is an active natural polyhydroxylated stilbene found in many plants. It has profound anti-inflammatory as well as antioxidant activities. However, it suffers relatively poor pharmacokinetic properties. Nanoformulation is an acknowledged approach to improve PIC bioavailability. The goal was to evaluate the ability of PIC in preventing testosterone-induced benign prostatic hyperplasia in rats. PIC was prepared in a self-nanoemulsifying drug delivery system (SNEDDS). Animals were placed into seven groups: 1) control (vehicle), 2) PIC SNEDDS (20 mg/kg), 3) testosterone (3 mg/kg), 4) testosterone + PIC SNEDDS (5 mg/kg), 5) testosterone + PIC (10 mg/kg), 6) testosterone + PIC SNEDDS (20 mg/kg) and 7) testosterone + finasteride (5 mg/kg). Testosterone was injected SC while PIC SNEDDS and finasteride were given orally. All treatments were given once daily, 5 days/week for four consecutive weeks. PIC administration ameliorated increased prostate weights and indices in addition to histopathological alterations. Further it inhibited accumulation of lipid peroxidation, depletion of glutathione (GSH) and exhaustion of catalase (CAT). PIC SNEDDS exhibited anti-proliferative activities as demonstrated by the inhibition of cyclin D1 protein expression and Bcl2 mRNA expression in addition to enhancement of Bax mRNA expression and caspase-3 content. Immunohistochemically, PIC SNEDDS protected against the testosterone-induced increased expression of tumor necrosis factor alpha (TNF-α), interleukin-6 (IL-6), cyclooxygenase-2 (COX-2), inducible nitric oxide synthase (iNOS), nuclear factor kappa B (NFκB) and also offered protection against the decline in Nrf2 expression. Further, a significant enhancement of Nfe212 and Homx1 mRNA expression was detected in PIC SNEDDS-treated animals in comparison to the testosterone group. Conclusively, PIC prepared in SNEDDS protects against experimentally induced BPH via modulation of, at least partly, Nrf2/HO-1/NFκB axis.

## Introduction

Benign prostatic hyperplasia (BPH) is a condition occurring mainly in middle-aged and older men (Berry et al., 1984). It is also a complication of several diseases including metabolic syndrome (Chughtai et al., 2016). Accumulating evidence indicates that metabolic syndrome and/or its components are associated with BPH (Li et al., 2020). Elevation in serum testosterone and insulin have been suggested to participate in BPH induced by metabolic syndrome (Muller et al., 2005). BPH includes an abnormally enlarged prostate gland that causes problematic symptoms, including: decreased urinary flow and frequency, trouble initiating flow, nocturia and dribbling (Berry et al., 1984; Devlin et al., 2020). Unfortunately, the histological prevalence of BPH at autopsy is believed to be as high as 50%–60% for men in their 60’s, and up to 80%–90% of those over 70 years of age (Ng and Baradhi, 2020). Although BPH has been studied extensively, actual triggers of the disease are yet to be determined (Berry et al., 1984). Hormonal and non-hormonal factors are thought to have essential roles in the pathophysiology of the disease (Culig et al., 1996; Cai et al., 2001; Escobar et al., 2009; Francisco and Francois, 2010; Wang and Olumi, 2011; Vikram and Jena, 2012). Underlying pathogenesis of the disease involves oxidative stress (Rogers et al., 1989) and inflammation (De Nunzio et al., 2016). Several factors can trigger an inflammatory response in the prostatic tissue. These include viral and bacterial infections, hormone imbalances, metabolic syndrome, diet or autoimmune diseases (De Nunzio et al., 2011; De Nunzio et al., 2016). Regardless of the initial trigger, inflammatory cells become activated and several cytokines are released leading to increased expression of different interferons and interleukins (De Nunzio et al., 2016). This in turn causes an irregular epithelial and stromal cell proliferation, with an increased oxygen demand (De Nunzio et al., 2016). An abundance of oxygen species (ROS) causes the development of oxidative stress (Shrilatha and Muralidhara, 2007). A causal role of oxidative stress and inflammation in prostate diseases has been previously reported (Paulis, 2018). Thus, oxidative stress remains among the main mechanisms that may account for BPH (Roumeguere et al., 2017). Main medical therapies of BPH include 5-α reductase inhibitors and adrenergic α-blockers (Lepor, 2011). However, these drugs can negatively influence the ejaculation process, cognitive functions and mental health (Kim et al., 2018). Because they are effective, less costly and well tolerated, phytotherapies are emerging as an additional and acceptable option for the management of BPH (Pagano et al., 2014).

Piceatannol (PIC) is a natural polyhydroxylated stilbene present in several foods such as grapes, blueberries, passion fruit seeds and peanuts (Kershaw and Kim, 2017). Many of these plants are widely cultivated in middle east countries (Diab et al., 2009; Giacalone et al., 2011). It is also a metabolite of resveratrol, a compound present in red wine grapes (Kershaw and Kim, 2017). However, PIC suffers relatively lower bioavailability when compared resveratrol (Setoguchi et al., 2014) and other stilbenes (Roupe et al., 2006). Therefore, nanoformulations have been utilized to improve pharmacokinetic and consequently pharmacodynamics characteristics of PIC (Tambuwala et al., 2019; Alhakamy et al., 2020; Aljabali et al., 2020). PIC offers beneficial effects in various conditions such as: hyperlipidemia, atherosclerosis, cardiovascular diseases and cancer (Kershaw and Kim, 2017). Its anti-proliferative and anti-invasive activities have been described (Kita et al., 2012; Alhakamy et al., 2020). It has been shown in the literature that PIC had strong free radical scavenging activity that was much higher than resveratrol (Murias et al., 2005). It has been postulated that PIC promotes anti-inflammatory signaling (Kershaw and Kim, 2017). The anti-inflammatory actions of PIC have been linked to heme oxygenase-1 (HO-1) and its activator nuclear factor erythroid 2-related factor (Nrf2) (Liu et al., 2011). In other studies, PIC was found to inhibit IL-6 and TNF-α expression, in addition to nuclear factor kappa beta (NF-κB) signaling in macrophages. It has also been reported to activate AMP-activated protein kinase (AMPK). The aim of this investigation was to determine the impact of a nanoformula of PIC on testosterone-induced BPH in rats and explore the possible mechanism(s) involved.

## Materials and Methods

### Chemicals

PIC was bought from Beijing Yibai Biotechnology. Co., Ltd. (Beijing, China) having over 98% purity. Testosterone enanthate from Chemical Industries Development Co. (CID), Giza, Egypt was used. Finasteride was obtained from (Merck KGaA, Darmstadt, Germany). Chemicals used in this investigation were of the highest analytical quality.

### Preparation of PIC Self-Nanoemulsifying Drug Delivery System (SNEDDS)

A SNEDDS was prepared by combining PIC with tween 80, PEG 200 and oleic acid (4:4:2) according to previous solubility studies. Components were mixed in their respective percentages totaling 1 g, were mixed with 50 mg PIC and placed in a vortex (5 min). This was followed by sonication using a Sonics probe sonicator (20 kHz, 1500 W, Sonics, Newtown, CT, United States) for 50 s to allow for the complete dissolution of PIC. PIC- SNEDDS globule size was determined by a dynamic light scattering technique utilizing a Nano-ZS particle size analyzer (Malvern Instrument, Worcestershire, United Kingdom), using 100 μL of PIC-SNEDDS, diluted with 10 ml of 0.1 N HCl, vortexed (1 min), then measured. Average globule size was 75 ± 2.1 nm.

### Animals

Procedures were approved by the Faculty of Pharmacy’s Research Ethics Committee, King Abdulaziz University (# PH-135–41). Male Wistar rats (195–220 g) of 10-weeks age were purchased from King Fahd Medical Research Center, King Abdulaziz University. An air-conditioned environment (22 ± 2°C) with alternating light/dark cycles was used for keeping the animals. Animals had a standard food pellet diet and water was accessed freely. Rodents were kept for 1 week in our facility to acclimatize them prior to starting the experimental protocol.

### Determination of Oral Lethal Dose 50 (LD50)

LD50 was determined in a separate experiment. A limiting dose of 2000 mg/kg of PIC SNEDDS was tried in three male rats and observed for 24 h. As no mortality was observed, the same procedure was repeated using additional three male rats; as guided by OECD (Organization for Economic Co-operation and Development) guideline no. 423 (OECD, 2002).

### Experimental Design

Forty two male Wistar rats were placed in seven groups randomly (6 rats per group). Group 1 was given a SC dose of maize oil (1 ml/kg) and oral plain vehicle of PIC SNEDDS (dosing volume of 10 ml/kg).

Group 2 was given a SC dose of maize oil (1 ml/kg) and oral PIC SNEDDS (20 mg/kg). Group 3 was injected with SC testosterone diluted in maize oil at a dose 3 mg/kg and oral plain vehicle (10 ml/kg). Testosterone (3 mg/kg, SC) and oral PIC SNEDDS (5, 10 or 20 mg/kg), were administered to groups 4, 5 and 6 respectively. Group 7 was also injected with SC testosterone and a daily oral dose of finasteride (5 mg/kg; dissolved in 2.5% ethanol) as a positive control. Oral administration was performed 1 h before SC injections. All treatments were given once daily, 5 days/week for four consecutive weeks. Chosen doses and schedules were based on a pilot experiment. At the end of the experiment, animals were euthenized using over dose of ether anesthesia. All efforts were done to minimize animal suffering. The prostate tissues were harvested after sacrificing the animals approximately 72 h post last treatment. A 10% neutral buffered formalin was used for storing parts of the prostatic ventral lobes for histopathological and immunohistochemical studies. The remaining prostatic tissues were placed momentarily in liquid nitrogen and kept at −80°C for assessing biochemical markers and mRNA expression using real-time polymerase chain reaction (RT-PCR). These tissues were used to assess prostate indices, histological alterations and oxidative status. Based on the obtained data, further experimentation was performed using tissues collected from animals in groups 1, 3, 4 and 5.

### Prostate Index and Weight

After dissection of the prostates, their weights were recorded and the prostate index was determined per animal by division of the weight of the prostate by the total body weight and multiplying it by 1,000.

### Histopathological Examination

Paraffin sections (4 μm thick) were made from the fixed prostatic tissues. Hematoxylin and eosin (H&E) was used to stain them after de-paraffinization and rehydration. Image J software (1.46a, NIH, United States) was employed in determining the height of the prostate glandular epithelia.

### Oxidative Status Markers Assessment

Ice cooled phosphate-buffered saline (50 mM potassium phosphate, pH 7.5) was used in prostate tissue homogenization. Homogenized samples were used to assess MDA, GSH, SOD, and total protein using commercially available kits (catalog # 10009055, 703002 and 707002 respectively, Cayman Chemical, Ann Arbor, MI, United States).

### Immunohistochemical Analyses

After deparrafinization, ethanol was used for rehydration of tissue sections. Sections were then subjected to boiling for 10 min in citrate buffer (pH 6.0). This was followed by a 2-h incubation in 5% bovine serum albumin (BSA) in tris buffered saline (TBS). Tissue sections were subsequently immersed at 4 ^o^C for 12 h with the primary antibodies. These were rabbit monoclonal anti-cyclin D1 (catalog # ab16663), polyclonal anti-IL-6 (catalog # ab271269), monoclonal anti-TNF-α (catalog # ab205587), polyclonal anti-COX-2 (catalog # ab15191), polyclonal anti-iNOS (catalog # ab3523), polyclonal anti-NFκB (p65) (catalog # ab16502) or polyclonal anti-Nrf2 (catalog # ab137550) (ABCAM, Cambridge, United Kingdom). TBS was used in slide flushing, followed by incubation with the biotinylated secondary antibody from a Cell and Tissue Staining Kit (Ant-rabbit HPRD-DAB system, catalog # CTS005, R&D systems, MN, United States). Image J analysis software (Image J, 1.46a, NIH, United States) was utilized in image analysis using at least three sections from each rat.

### Real-Time Polymerase Chain Reaction (RT-qPCR) Analysis of Bax, Bcl-2 Nfe2l2 and Hmox1

Prostates were homogenized in an ultrasonic probe. A nucleic acid extraction kit (NucleoSpin, REF # 740955) bought from Macherey-Nagel GmbH and Co. KG, Duerin, Germany was used to extract RNA. A spectrophotomer (dual-wavelength Beckman, Spectrophotometer, United States) was used to assess the purity (A260/A280 ratio) and RNA concentration. A cDNA Reverse Transcription Kit (catalog # 4368814, Applied Biosystems, Foster City, CA, United States) was utlized for reverse transcription. A Taq PCR Master Mix Kit (catalog # 201443, Qiagen, Valencia, CA, United States) was employed for PCR amplification. Expression of Bax, Bcl2, Nfe2l2, Hmox1 was assessed relative to β-actin. Sequence of the used forward and reverse primers of each gene is illustrated in Table 1. After the RT-PCR run, the data were given in the cycle threshold (Ct). The relative quantitation (RQ) of the different genes to β-actin was determined based on determining delta-delta Ct (ΔΔCt).

**TABLE 1:** Primers sequences.

	Forward	Reverse
Bax	CCT​GAG​CTG​ACC​TTG​GAG​CA	GGT​GGT​TGC​CCT​TTT​CTA​CT
Bcl2	TGA​TAA​CCG​GGA​GAT​CGT​GA	AAA​GCA​CAT​CCA​ATA​AAA​AGC
Nfe2l2	TCC​CAA​ACA​AGA​TGC​CTT​GT	AGA​GGC​CAC​ACT​GAC​AGA​GA
Hmox1	CAT​CCG​TGC​AGA​GAA​TTC​TG	CTG​GTA​TGG​GCC​CCA​CTG​GC
β-actin	TCC​GTC​GCC​GGT​CCA​CAC​CC	TCA​CCA​ACT​GGG​ACG​ATA​TG

### Determination of Caspase-3

ELISA kit (product # SEA626Ra, USCN Life Science Inc., China) was employed in determining the content of caspase-3 in the prostate homogenate.

### Assessment of Protein Content

A commercial kit based on bicinchoninic acid (BCA) for the colorimetric quantitation of total protein in prostate homogenates was used (catalog # 23,225, Thermo Fisher Scientific, Waltham, MA, U.S).

### Statistical Analysis

Data are presented as mean ± SD. One-way ANOVA and Tukey’s post hoc test were used for multiple comparisons. Statistical significance was taken at *p* < 0.05. GraphPad Prism version 8 (GraphPad, La Jolla, CA, United States) was employed for statistical tests.

## Results

### Acute Oral Toxicity Study

A single oral dose of PIC SNEDDS (2000 mg/kg) resulted in no mortality at 24 h after administration to three rats. The same observation was recorded when the test was repeated using three additional animals at the same dose level. According to the Acute Toxic Class Method reported in OECD guidelines No.423, PIC SNEDDS is considered to be Category 5 with LD50 > 2000 mg/kg.

### Prostate Weights and Indices

As indicated by the data in Table 2, PIC SNEDDS (20 mg/kg) did not cause any significant effects on prostate weight. However, testosterone injection significantly increased prostate weights and indices by 200% and 174% respectively, relative to the corresponding control group. Animals exhibited a notable decrease in weights of the prostate by 30%, 55%, and 57% the prostate index by 27%, 53%, and 54%, respectively with PIC SNEDDS co-treatment at 5, 10 and 20 mg/kg relative to the testosterone group. Finasteride prevented the rise in prostate weight and index by and 62% and 58%, respectively as compared to testosterone group. It is noteworthy to report that PIC SNEDDS (20 mg/kg) and finasteride did not exhibit significant effects when compared to PIC SNEDDS (10 mg/kg).

**TABLE 2:** Effect of PIC on prostate weight and prostate index in testosterone-induced BPH in rats.

	Body weight (g)	Prostate weight (g)	Prostate index X10^3^
Control	271.7 ± 15.04	0.25 ± 0.03	0.93 ± 0.13
PIC (20 mg/kg)	267.67 ± 10.80	0.25 ± 0.04	0.94 ± 0.17
Testosterone (test)	295.0 ± 14.15	0.75^a^ ^,^ ^b^ ± 0.11	2.55^a^ ^,^ ^b^ ± 0.33
Test + PIC (5 mg/kg)	282.7 ± 15.36	0.52^a^ ^,^ ^b^ ^,^ ^c^ ± 0.05	1.85^a^ ^,^ ^b^ ^,^ ^c^ ± 0.24
Test + PIC (10 mg/kg)	267.3 ± 13.1	0.34^c^ ^,^ ^d^ ± 0.05	1.21^c^ ^,^ ^d^ ± 0.17
Test + PIC (20 mg/kg)	270.00 ± 7.87	0.32^c^ ^,^ ^d^ ± 0.04	1.17^c^ ^,^ ^d^ ± 0.17
Test + finasteride (5 mg/kg)	262.67 ± 8.58	0.28^c^ ^,^ ^d^ ± 0.05	1.07^c^ ^,^ ^d^ ± 0.23

Prostate index = Prostate weight/Body weight ratio. Data are expressed as mean ± SD.

a, b, c, d : statistically different from corresponding control, PIC (20 mg/kg), testosterone or Test + PIC (5 mg/kg) respectively, at *p* < 0 05.

### Histopathological Examination

**FIGURE 1:**
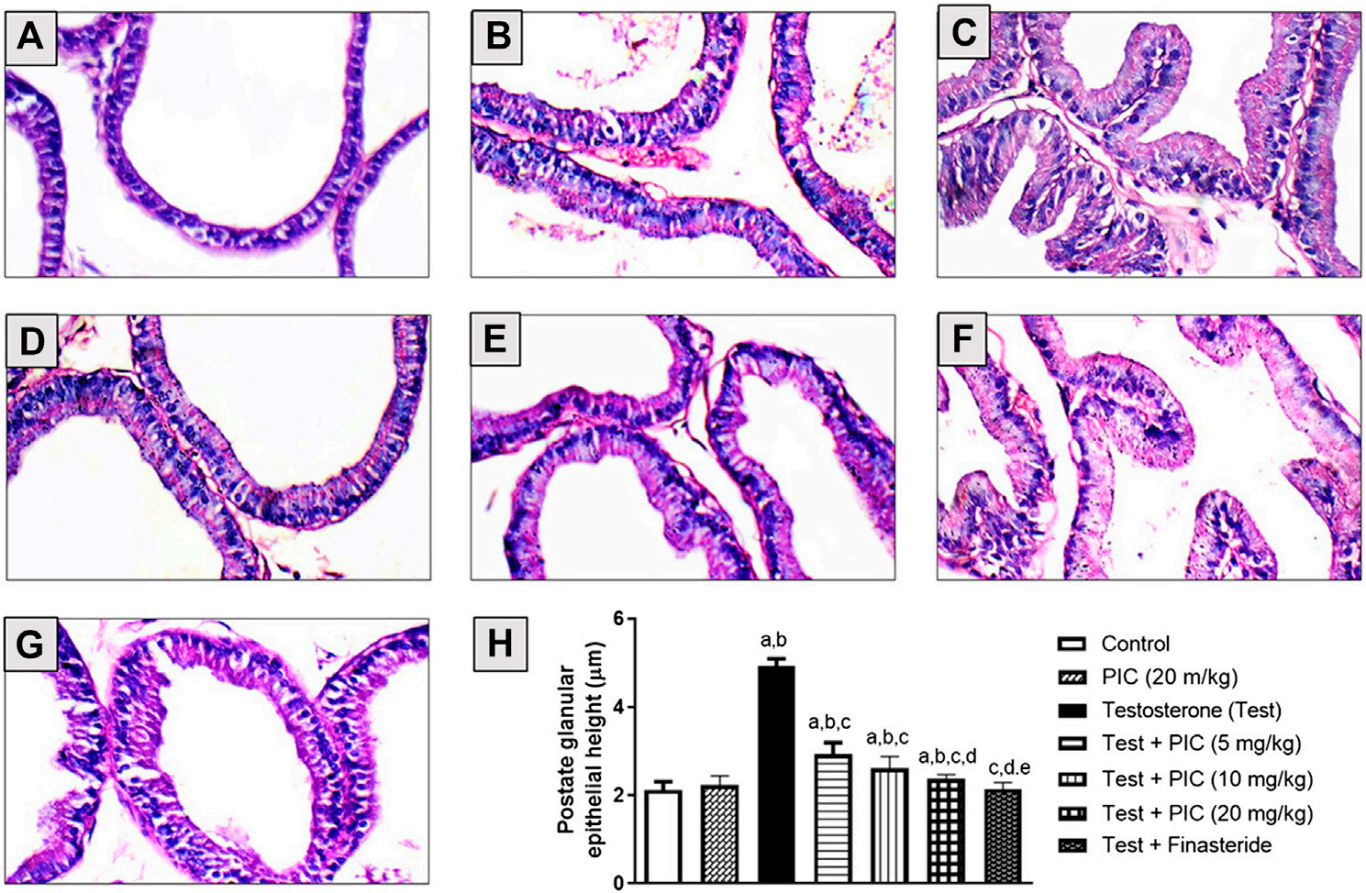
Hematoxylin-eosin stained sections of rat prostates. **(A)** The normal histoarchitecture of the prostate gland (control group). **(B)** Section for prostatic tissues obtained from PIC SNEDDS (20 mg/kg) alone with no observable histological observations. **(C)** Prostate section from testosterone-treated animals with increased epithelial thickness and hypertrophy (testosterone group). **(D)** Prostate section from animals co-treated with PIC SNEDDS (5 mg/kg) with a slight reduction in hyperplasia and hypertrophy. **(E)** Prostate section from animals co-treated with PIC SNEDDS (10 mg/kg) with a clear reduction in hyperplasia and hypertrophy. **(F)** Prostate section obtained from PIC (20 mg/kg) with much less hyperplasia. **(G)** Prostate section from finasteride (5 mg/kg) group with observable protection against testosterone-induced hyperplasia. **(H)** Figure showing the prostate glandular epithelial height with PIC co-treatment. Results are shown as mean ± SD (*n* = 6). Data are expressed as mean ± SD; *a*, *b*, *c*, *d* or *e*: statistically different from corresponding control, PIC SNEDDS (20 mg/kg), testosterone, Test + PIC SNEDDS (5 mg/kg) or Test + PIC SNEDDS (10 mg/kg) respectively, at *p* < 0 05. using one-way ANOVA followed by Tukey’s post-hoc test.

Histopathological examinations of prostatic tissues obtained from control and PIC SNEDDS alone (20 mg/kg)-treated animals indicated average acini lined by single layer of cuboidal epithelial cells with basally-located nuclei, intact basement membrane and average stroma (Figures 1A,B). Testosterone injection resulted in markedly hyperplastic acini lined by single layer of tall columnar epithelial cells with nuclear pseudostratification, papillary projections, and hypertrophied stroma (Figure 1C). However, prostates of animals administered PIC SNEDDS (5, 10 and 20 mg/kg) showed much less hyperplastic growth evidenced by average acini and stroma in a dose-dependent fashion (Figures 1D–F). Comparable protective effects were observed in the finasteride-treated animals (Figure 1G). These data were confirmed by assessing glandular epithelial height. PIC SNEDDS (5, 10 and 20 mg/kg) and finasteride significantly prevented the increase in epithelial height by 40%, 47%, 51% and 56% respectively relative to the testosterone group (Figure 1H).

### Markers of Oxidative Stress

As shown in Table 3, PIC SNEDDS alone at a dose of 20 mg/kg did not alter any of the oxidative status markers. In the testosterone-alone group, MDA levels were increased by two fold and the GSH content and CAT activity were reduced by 58% and 46% respectively relative to the controls. However, administration of PIC SNEDDS (5, 10 and 20 mg/kg) caused a considerable improvement in the testosterone-related rise in MDA lipid peroxidation by 53%, 66% and 67% respectively. Furthermore, the middle PIC SNEDDS dose (10 mg/kg) guarded against GSH depletion and CAT exhaustion and boosted their values by 135% and 75% respectively. Finasteride significantly ameliorated the rise in MDA content, depletion of GSH and exhaustion of CAT in prostatic tissues.

**TABLE 3:** Effect of PIC on oxidative status in testosterone-induced BPH in rats.

	MDA (nmol/mg protein)	GSH (nmol/mg protein)	CAT (U/mg protein)
Control	0.522 ± 0.066	1.70 ± 0.13	0.840 ± 0.049
PIC (20 mg/kg)	0.517 ± 0.061	1.83 ± 0.14	0.874 ± 0.060
Testosterone (test)	1.619^a^ ^,^ ^b^ ± 0.203	0.70^a^ ^,^ ^b^ ± 0.06	0.452^a^ ^,^ ^b^ ± 0.059
Test + PIC (5 mg/kg)	0.757^a^ ^,^ ^b^ ^,^ ^c^ ± 0.036	1.33^a^ ^,^ ^b^ ^,^ ^c^ ± 0.38	0.615^a^ ^,^ ^b^ ^,^ ^c^ ± 0.044
Test + PIC (10 mg/kg)	0.544^a^ ^,^ ^b^ ^,^ ^c^ ± 0.045	1.65^c^ ± 0.16	0.792^c^ ^,^ ^d^ ± 0.080
Test + PIC (20 mg/kg)	0.532^a^ ^,^ ^b^ ^,^ ^c^ ± 0.057	1.60^c^ ± 0.17	0.772^c^ ^,^ ^d^ ± 0.087
Test + finasteride (5 mg/kg)	0.611^a^ ^,^ ^b^ ^,^ ^c^ ± 0.074	1.53^c^ ± 0.17	0.758^c^ ^,^ ^d^ ± 0.094

Data are expressed as mean ± SD.

a, b, c, d : statistically different from corresponding control, PIC (20 mg/kg), testosterone or Test + PIC (5 mg/kg) respectively, at *p* < 0 05.

## Anti-Proliferative Activities of PIC

### Cyclin D1

**FIGURE 2:**
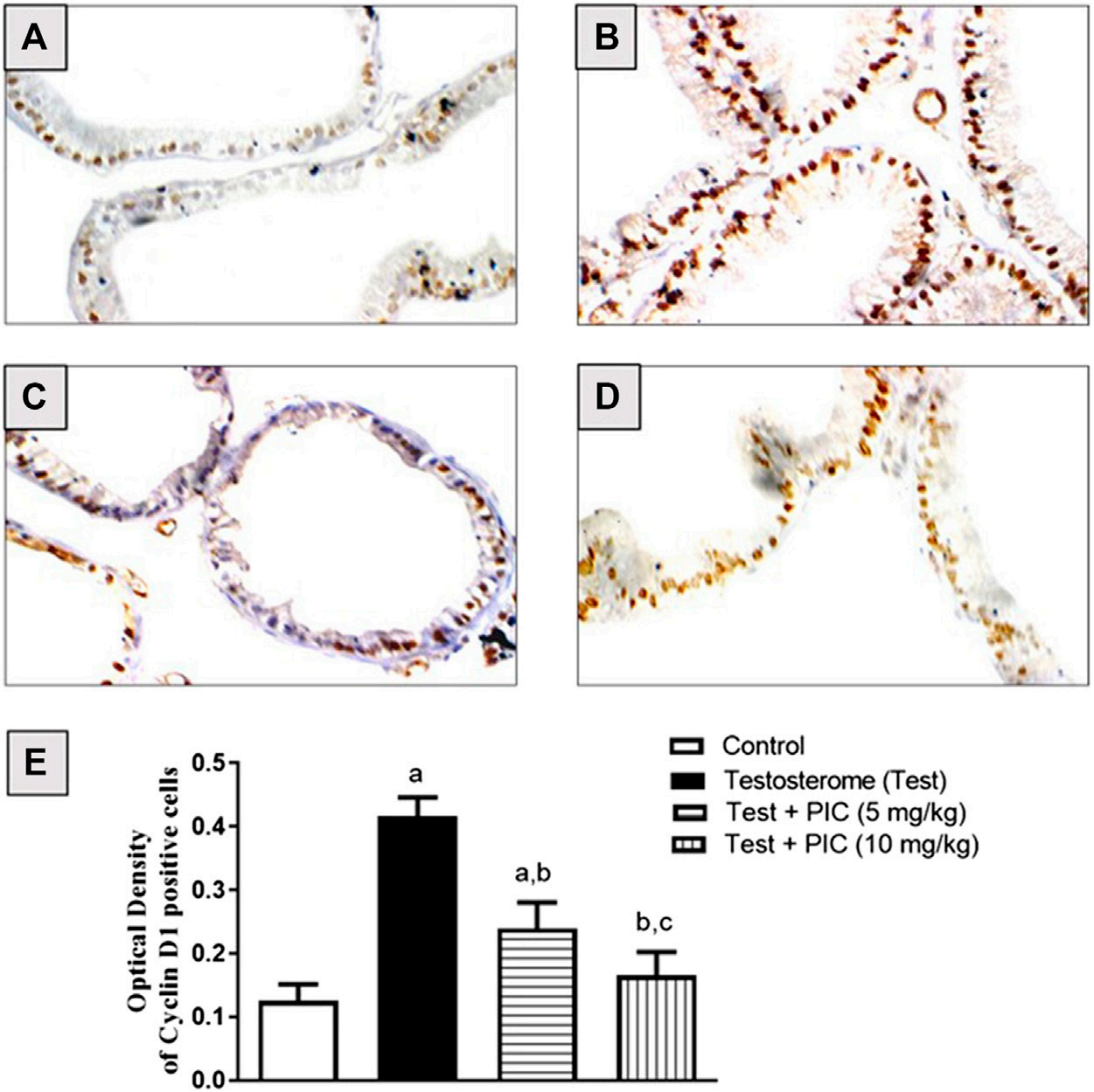
Immunohistochemistry photomicrographs of prostate sections showing the influence of PIC SNEDDS on cyclin D1 expression in rats treated with testosterone. **(A)** Prostate section from control rats, **(B)** Prostate section from rats treated with testosterone. **(C)** Prostate section from rats treated with testosterone + 5 mg/kg PIC SNEDDS. **(D)** Prostate section from rats treated with testosterone + 10 SNEDDS mg/kg PIC. **(E)** The effect of PIC SNEDDS co-treatment on cyclin D1 prostate expression. Results are shown as mean ± SD (*n* = 6). *a*, *b* or *c*: Statistically different from control, testosterone or PIC SNEDDS (5 mg/kg) group, respectively at *p* < 0.05 using one-way ANOVA followed by Tukey’s post-hoc test.

Cyclin D1 protein expression was determined using immunohistochemistry and it was revealed that the control group had moderately stained cells (Figure 2A). Testosterone injection caused a higher intensity of stained cells highlighting a higher rate of proliferation (Figure 2B). PIC SNEDDS (5 and 10 mg/kg) co-treatment significantly lowered the intensity of cells positive for cyclin D1 in comparison to the testosterone group in a dose-related manner (Figures 2C,D). Cells positive for cyclin D1 were densitometrically quantified. Results in Figure 2E indicate that PIC SNEDDS (5 and 10 mg/kg) resulted in lowered expression of cyclin D1 by 41% and 61%, respectively.

### mRNA Expression of Bax and Bcl-2 

**FIGURE 3:**
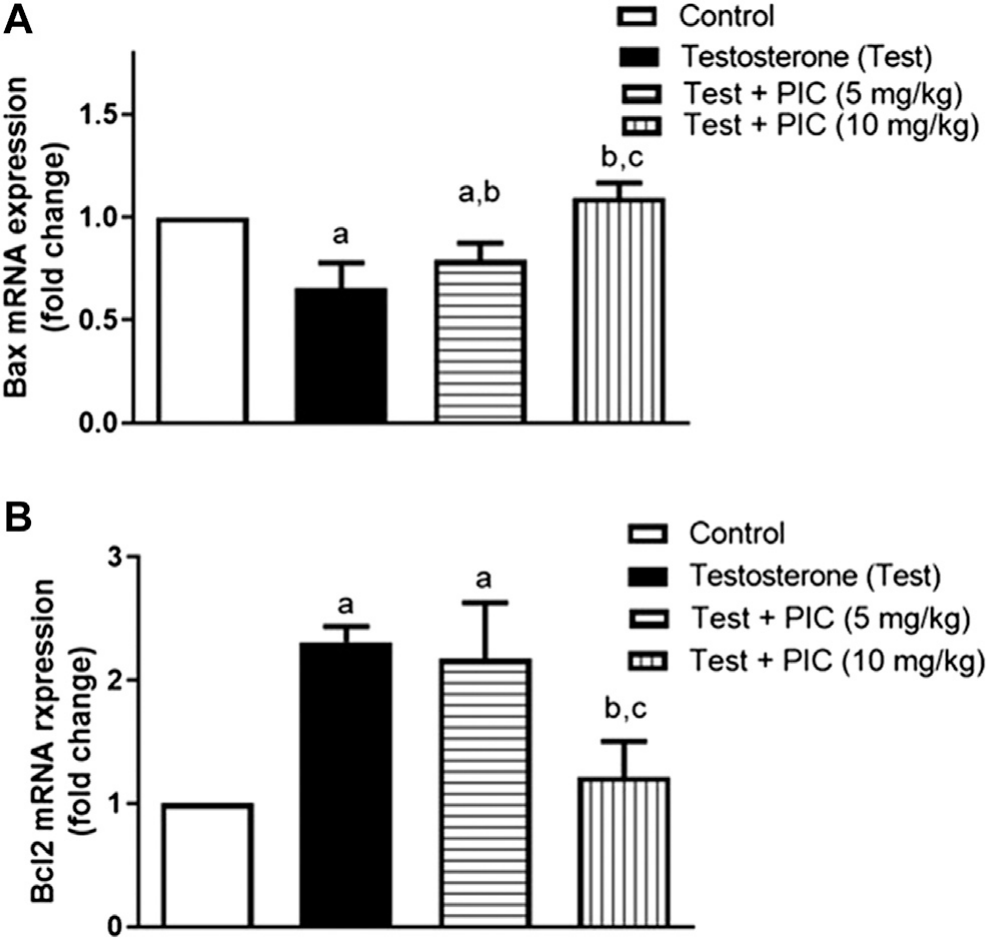
Effects of PIC SNEDDS treatment on mRNA levels of Bax (Panel **(A)**) and Bcl-2 (Panel **(B)**) in the prostate gland. Results are shown as the mean ± S. D (*n* = 6). *a*, *b* or *c*: Statistically different from control, testosterone or PIC SNEDDS (5 mg/kg) group, respectively at *p* < 0.05 using one-way ANOVA followed by Tukey’s post-hoc test.

Testosterone significantly reduced Bax mRNA expression by 35% in comparison to controls. On the other hand, PIC SNEDDS (5 and 10 m/kg) resulted in a significant protection from the testosterone-induced Bax mRNA expression reduction by 14 and 35% of the control values, respectively (Figure 3A). The testosterone group showed enhanced expression of Bcl2 mRNA by 130% as compared to control group. PIC SNEDDS (5 and 10 mg/kg) significantly prevented this rise by 6% and 47% respectively, compared to testosterone group (Figure 3B).

### Caspase 3

**FIGURE 4:**
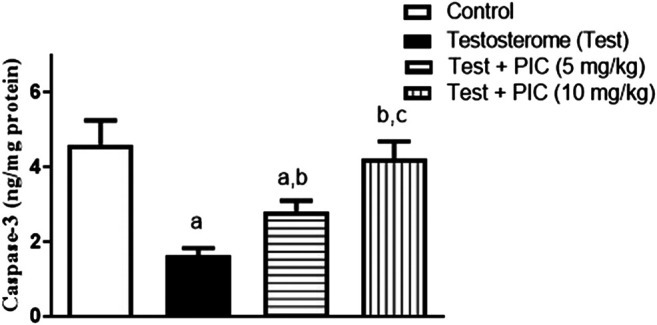
Effects of PIC SNEDDS treatment on caspase-3 content in the prostate gland. Results are shown as the mean ± S.D (*n* = 6). *a*, *b* or *c*: Statistically different from control, testosterone or PIC SNEDDS (5 mg/kg) group, respectively at *p* < 0.05 using one-way ANOVA followed by Tukey’s post-hoc test.

As shown in Figure 4, testosterone injection resulted in significant decrease in caspase-3 content of prostatic tissues by 65% as compared to control value. However, PIC SNEDDS (5 mg/kg) significantly enhanced caspase-3 content by 75% as compared to testosterone group. PIC SNEDDS (10 mg/kg) efficiently prevented the decline in caspase-3 content and almost normalized its value.

### Immunohistochemical Determination of Prostate Expression of IL-6, TNF-α, COX-2, iNOS, NFκB (p65) and Nrf2

**FIGURE 5:**
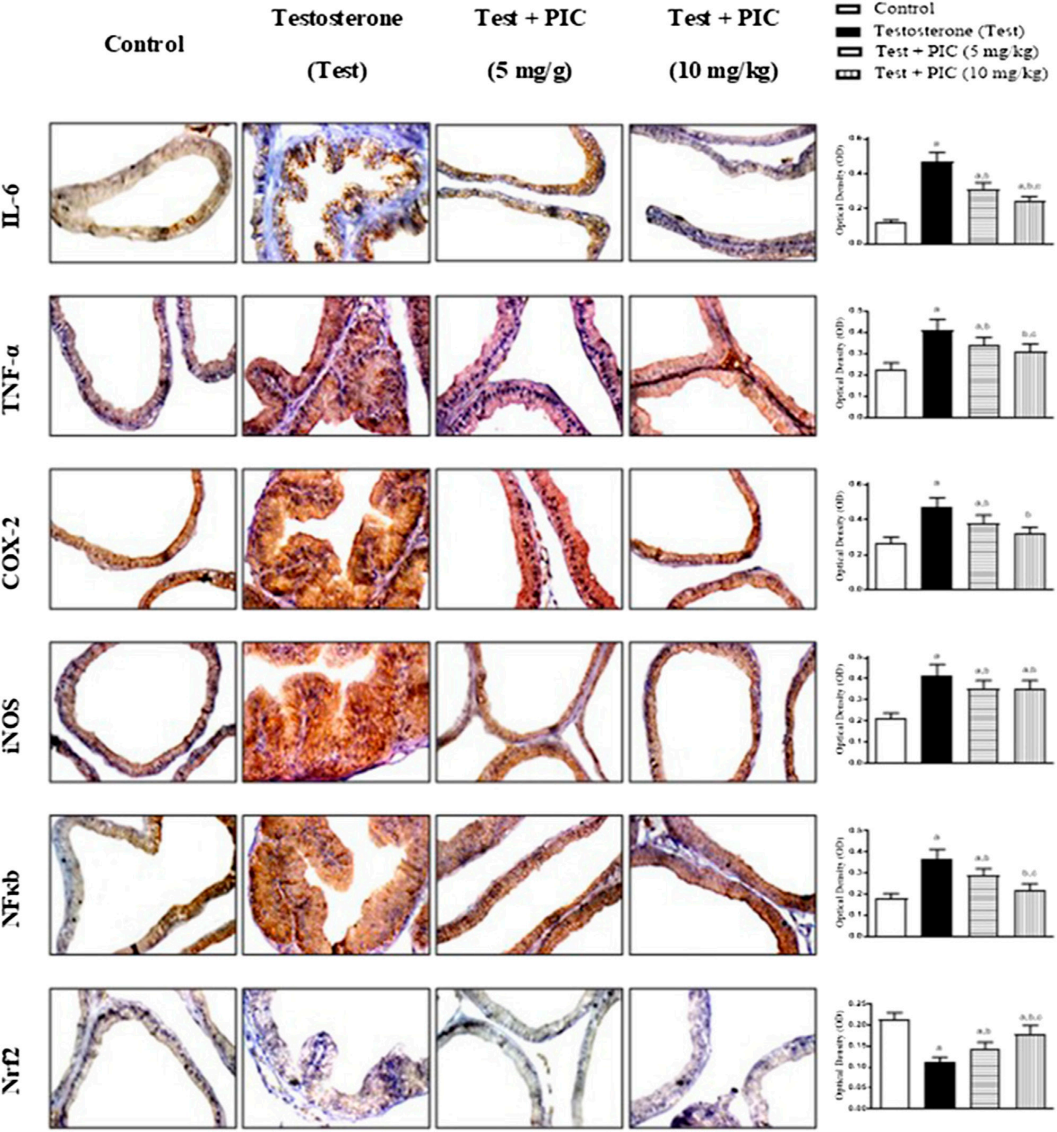
Photomicrographs of prostate sections immunohistochemically demonstrating the effect of PIC SNEDDS on testosterone-induced alteration of IL-6, TNF-α, COX-2, iNOS, NFκB and Nrf2 expression in the prostate gland. Data presented in bar charts are mean of optical densities ±S. D (*n* = 6). *a*, *b* or *c*: Statistically different from control, testosterone or PIC SNEDDS (5 mg/kg) group, respectively at *p* < 0.05 using one-way ANOVA followed by Tukey’s post-hoc test.

As shown in Figure 5, testosterone injection caused a marked rise in inflammatory markers’ IL-6, TNF-α, COX-2 and iNOS expression by 275%, 78%, 74% and 95% respectively, of the control value. Examination of NFκB indicated that testosterone also enhanced its densities in the assessed nuclei by almost double the control value. However, PIC SNEDDS (5 and 10 mg/kg) led to a significant protection against the rise of expressed TNF-α, IL-6, COX-2, iNOS and NFκB (p65). On the other hand, testosterone exposure resulted in a significant decline in Nrf2 to almost half the control value. At dose levels of 5 and 10 mg/kg, PIC SNEDDS had a significant protection with regards to the decline in Nrf2 expression and boosted its values by 27% and 54% of the testosterone values.

### Assessment of mRNA Expression of Nfe212 and Homx1

**FIGURE 6:**
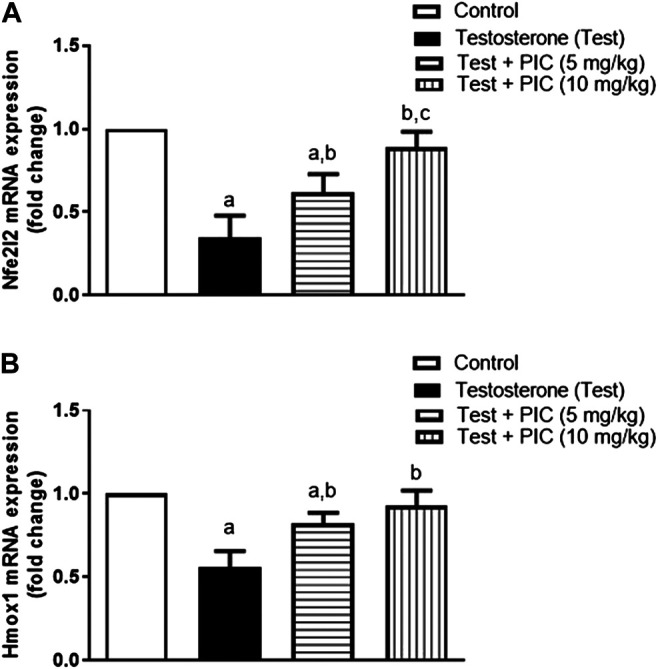
Effects of PIC SNEDDS treatment on mRNA levels of Nfe212 (Panel **(A)**) and Homx1 (Panel **(B)**) in the prostate gland. Results are shown as the mean ± S. D (*n* = 6). *a*, *b* or *c*: Statistically different from control, testosterone or PIC SNEDDS (5 mg/kg) group, respectively at *p* < 0.05 using one-way ANOVA followed by Tukey’s post-hoc test.

To further substantiate the role of Nrf2, prostatic expression of Nfe212 mRNA was assessed. As shown in Figure 6A, testosterone injection resulted in a notable reduction of the gene expression by 65% of the control value. However, co-treatment with PIC SNEDDS at doses of 5 and 10 mg/kg has a significant enhancement of Nfe212 expression by 79% and 158% as compared to testosterone group. Also, mRNA expression of the downstream Homx1 was assessed. Figure 6B indicates that testosterone caused a parallel downregulation of Homx1 expression amounting to 45% of the control value. However, both the lower dose of PIC SNEDDS could significantly ameliorate the decline in Homx1 expression to 82% of the control values. The higher dose of PIC SNEDDS almost normalized Homx1 mRNA expression.

## Discussion

BPH is well-recognized as a health hazard in middle-aged and elderly men (Donnell, 2011). Although benign, this condition is characterized by an enlarged prostate resulting from a hyper-proliferative state in the stromal, glandular and mesenchymal prostate cells (Roehrborn, 2011). This causes a number of lower urinary tract symptoms, that can ultimately deteriorate a patient’s quality of life. BPH pathogenesis is linked to oxidative stress and imbalanced cell proliferation and apoptosis (Pawlicki et al., 2004; Minciullo et al., 2015). PIC is a natural stilbene with known anti-oxidant, anti-proliferative and anti-inflammatory properties (Banik et al., 2020). The aim of this study was to evaluate the potential of PIC to prevent testosterone-induced BPH in rats and explore the possible mechanism(s) involved. In the current study, PIC was prepared as SNEDDS so as to improve its reported poor solubility and bioavailability (Tambuwala et al., 2019). Additionally, the prepared PIC SNEDDS exhibited a highly non-toxic profile as evidenced by the oral LD50 testing which showed that the preparation is considered to be Category 5 with LD50 > 2000 mg/kg. It significantly hampered testosterone-related increases in prostate weights, hyperplastic growth of acini and stroma as well as epithelial heights. This agrees with previously reported anti-proliferative activity in different types of cell lines including prostate cancer cells (Kuo and Hsu, 2008; Kita et al., 2012; Kwon et al., 2012; Zheng and Wu, 2020). Further, exposure to testosterone resulted in oxidative stress induction in prostatic tissues. Actually, the role of oxidative stress in BPH has been shown and well-documented (Udensi and Tchounwou, 2016). However, PIC SNEDDS protected against the generated oxidative stress in prostatic tissues. This was evidenced by the ability of PIC SNEDDS to inhibit the rise in lipid peroxidation products, GSH depletion and CAT exhaustion. These data also agree with the known antioxidant actions of PIC (Sochorova et al., 2020). PIC antioxidant properties can be attributed to its chemical structure. The compound demonstrates poly phenolic groups that can form semiquinone and stabilize reactive oxygen radicals and thus exert anti-oxidation (Piotrowska et al., 2012). Taking the data of the initial dose-response study, it can be deduced that PIC SNEDDS are highly safe and the higher dose (20 mg/kg) did afford extra protection to prostatic tissues as compared the middle dose (10 mg/kg). Therefore, PIC SNEDDS (5 and 10 mg/kg) were used for subsequent experimentation in comparison to control and testosterone-alone treated animals.

The anti-inflammatory of PIC SNEDDS in prostatic tissues was confirmed immunohistochemically. It was observed that prostatic hyperplasia was accompanied and/or preceded by a background of inflammatory status. Studies on the pathophysiology of BPH highlighted a significant role for inflammation and inflammatory mediators (McClinton et al., 1990; Theyer et al., 1992; Madersbacher et al., 2019; Devlin et al., 2020). In particular, inflammation was reported to be a key-player in testosterone-induced BPH (Rastrelli et al., 2019). PIC SNEDDS significantly prevented the rise in expression of IL-6, TNF-α, COX-2, iNOS and NFκB (p65). This is supported by the known anti-inflammatory effects of PIC and all stilbene compounds (Dvorakova and Landa, 2017). Also, these data support the observed antioxidant activities of PIC based on the reported inflammation-mediated oxidative stress in male genital tissues (Agarwal et al., 2018). Thus, it can be concluded that PIC SNEDDS anti-inflammatory properties significantly contribute to the observed anti-hyperplasia effects.

Initiation of inflammation in prostatic micro-environment can lead to imbalanced cellular proliferation and apoptosis causing stromal and glandular hyperplasia (Chughtaiet al., 2016). Thus, impact of the observed PIC SNEDDS anti-inflammatory activities on proliferation and apoptosis was assessed in the current study. Cyclin D1 is involved in regulating cell proliferation and its abundance is commonly interpreted in favor of cell division (Fu et al., 2004). In line with previous studies (Abdel-Naim et al., 2018; Basha et al., 2019), our data indicated that testosterone significantly enhanced cyclin D1 expression. However, PIC SNEDDS successfully hampered the raised cyclin D1 expression as detected immunohistochemically. B-cell lymphoma-2 (Bcl-2) proteins are known to regulate this balance by inhibiting apoptosis, while Bax proteins increase apoptosis. They both therefore exhibit opposing activities, whereby Bcl-2 stops while Bax increases cytochrome c release, which is responsible for caspase-3 activation leading to apoptosis (Fischer et al., 2003). An anti-apoptotic effect of testosterone was seen in the prostate gland by causing an upregulated Bcl-2 and a downregulated Bax, thereby lowering cytochrome c levels and activating caspase-3 (Shukla et al., 2007). Our results showed that PIC SNEDDS treatment lowered Bcl-2 and enhanced expression of Bax mRNA in prostatic tissues of animals challenged with testosterone. Further, the reduction in caspase-3 levels induced by testosterone was reversed by the administration of PIC SNEDDS, whereby the enzyme contents were almost normalized by the higher dose.

The Nrf2/HO-1 pathway has gained much attention recently with regards to its involvement in inflammation resulting from NFκB activation (Wardyn et al., 2015). Physiologically, Kelch-like ECH-associated protein-1 (Keap1) is kept stable by the binding of Nrf2. Nrf2 enters the nucleus after dissociating from Keap1 whereby it binds to anti-oxidant-responsive elements. This causes downstream genes such as HO-1 to become upregulated and alleviates inflammation by affecting p65 translocation (Wardyn et al., 2015). It has been demonstrated that PIC SNEDDS increased HO-1 expression due to its ability to activate Nrf2 in various cell lines including MCF10A epithelial cells (Lee et al., 2010) as well as bovine endothelial cells (Wung et al., 2006). Furthermore, PIC SNEDDS was found to increase mRNA expression of Nrf2 and HO-1 as well as Nrf2 nuclear translocation in an animal model of diabetic cardiomyopathy (Li et al., 2019). In this investigation, testosterone inhibited protein expression of Nrf2 in prostate tissues. However, this was prevented by the administration of PIC SNEDDS. This was confirmed by the observed ability PIC SNEDDS to inhibit lowered mRNA expression of Nfe212 and Hmox1 due to testosterone. These observations align with the observed antioxidant and anti-inflammatory properties of PIC SNEDDS. Finally, PIC SNEDDS protected against experimentally-induced BPH via modulation of, at least partly, Nrf2/HO-1/NFκB axis.

## Data Availability Statement

The raw data supporting the conclusions of this article will be made available by the authors, without undue reservation.

## Ethics Statement

The animal study was reviewed and approved by Research Ethics Committee, Faculty of Pharmacy, King Abdulaziz University (# PH-135-41).

## Author Contributions

All authors listed have made a substantial, direct, and intellectual contribution to the work and approved it for publication.

## Funding

This project was funded by the Deanship of Scientific Research (DSR) at King Abdulaziz University, Jeddah, under Grant no. G: 282-166-1441. The authors, therefore, acknowledge with thanks DSR for the technical and financial support.

## Conflict of Interest

The authors declare that the research was conducted in the absence of any commercial or financial relationships that could be construed as a potential conflict of interest.
