# Supplementary figures and images for: Piceatannol Attenuates Testosterone-Induced Benign Prostatic Hyperplasia in Rats by Modulation of Nrf2/HO-1/NFκB Axis
Source: Front Pharmacol. 2020 Dec 21;11:614897. doi: 10.3389/fphar.2020.614897 (PMC7845651; doi:10.3389/fphar.2020.614897)

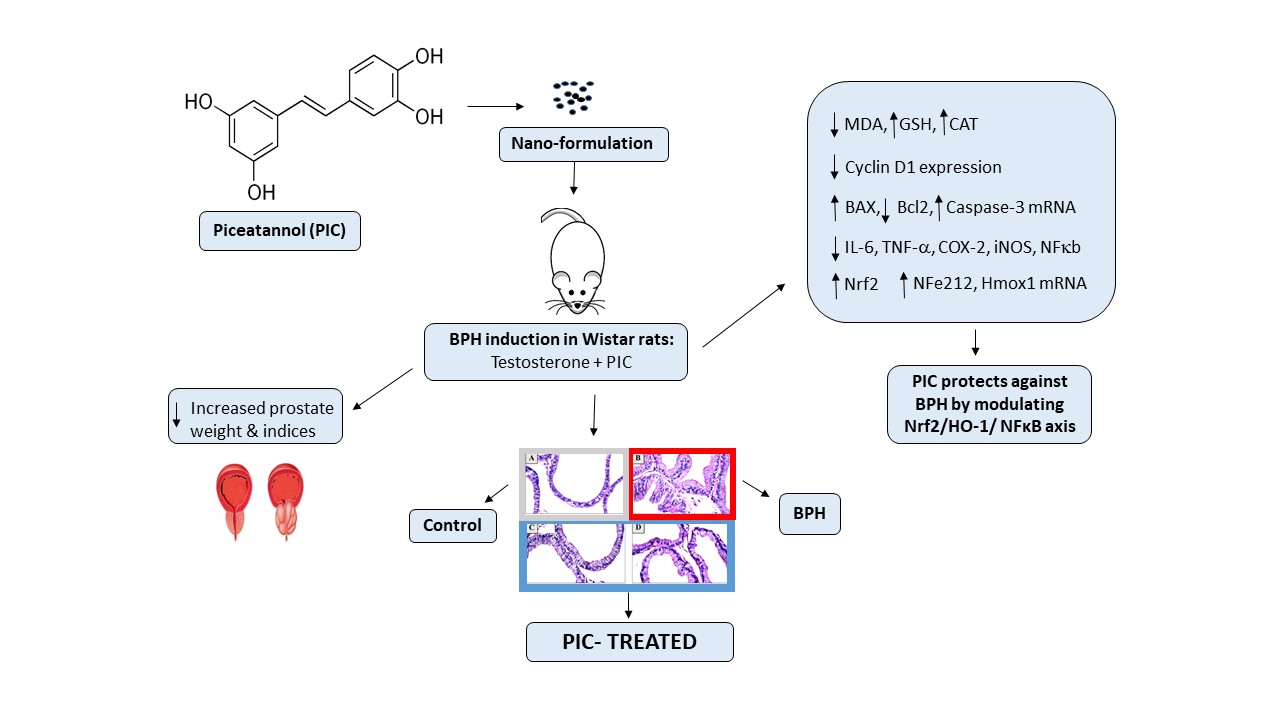

Supplement: Supplementary file 1 [file image1.jpeg]
